# Association between polymorphisms of *MIR17HG *and risk of colorectal cancer in the Chinese Han population

**DOI:** 10.1002/mgg3.667

**Published:** 2019-04-03

**Authors:** Peng Chen, Yuwei Bai, Yaru Li, Yuemin Yuan, Yimin Cheng, Jianjian Pang, Hongli Zhu, Chao Chen

**Affiliations:** ^1^ The National Engineering Research Centre for Miniaturized Detection Systems College of Life Science Northwest University Xi’an P.R. China; ^2^ Institution of Basic Medical Science Xi’an Medical University Xi’an P.R. China; ^3^ Xi’an Chest Hospital Xi’an P.R. China

**Keywords:** case–control study, colorectal cancer, *MIR17HG*, single nucleotide polymorphisms, susceptibility

## Abstract

**Background:**

Colorectal cancer is the third most common cancer worldwide. Recently, an increasing number of evidences suggest that genetic susceptibility plays an important role in the occurrence of colorectal cancer. This study aimed to better understand the influence of *MIR17HG* polymorphisms on colorectal cancer susceptibility in the Chinese Han population.

**Methods:**

We recruited 514 patients with colorectal cancer and 510 healthy controls to investigate the association between polymorphisms of *MIR17HG* and risk of colorectal cancer in the Chinese Han population. Genotyping was performed with the Agena MassARRAY platform. We used the *χ* ^2^ test to compare the distributions of single nucleotide polymorphisms (SNPs) allele and genotypes frequencies between cases and controls. Odds ratios and 95% confidence intervals were calculated by logistic regression analysis to evaluate the association under genetic models. Linkage disequilibrium between the five SNPs was assessed using the Haploview software.

**Results:**

Overall analysis found that rs7336610 and rs1428 and haplotype CTAGA were significantly associated with increased risk of colorectal cancer. However, we found rs7318578 was associated with a decreased risk of colorectal cancer in the dominant model. Stratification analysis showed that rs7336610, rs7318578, and rs1428 were also associated with rectal cancer risk. Gender stratification analysis found that rs7336610, rs7318578, rs17735387, and rs1428 were significantly associated with colorectal cancer risk in males.

**Conclusion:**

In conclusion, this study indicated that the polymorphisms of *MIR17HG* were associated with colorectal cancer risk. Therefore, our findings may provide new insights into the development of colorectal cancer. Further association and functional studies are of great importance to confirm these results and to define the potential biological mechanism of colorectal cancer.

## 1 INTRODUCTION

Colorectal cancer is the third most common cancer worldwide and a major causes of cancer related morbidity and mortality (Bray et al., 2018). In China, the incidence and mortality of colorectal cancer have a rapid increase during the past few decades (Chen et al., 2016). The colorectal cancer occurrence and progression are comprehensive, multifactorial, and multistep process which caused by the interaction of environmental and genetic factors. However, the mechanism of colorectal carcinogenesis remains still not fully understood. Although obesity, sedentary behavior, and a high‐meat, high‐calorie, fat‐rich, fiber‐deficient diet, alcohol consumption, and tobacco smoking were found to be major risk factors for the development of colorectal cancer (Bishehsari, Mahdavinia, Vacca, Malekzadeh, & Mariani‐Costantini, 2014; Marley & Nan, 2016), only a fraction of individuals exposed to the same risk factors develop colorectal cancer during their lifetime, suggesting that other factors were associated with the development of colorectal cancer. The single nucleotide polymorphism (SNP) is the most common form of human genetic variations and are significantly associated with many cancers risk (Geng et al., 2015; Hu et al., 2019; Tian et al., 2018). Recently, an increasing number of evidence suggests that genetic susceptibility plays an important role in the occurrence of colorectal cancer (Duan et al., 2014; Su et al., 2015; Wang et al., 2015; Zhang, Li, Du, et al., 2014b).

The miR‐17‐92a‐1 cluster host gene (MIR17HG) located on chromosome 13q31.3 in the third intron of an open reading frame termed the *c13orf25 *(chromosome 13 open reading frame 25) gene. The miR‐17‐92 cluster transcript is about 800 nucleotides and encompasses six miRNAs (miR‐17, miR‐18a, miR‐19a, miR‐20a, miR‐19b‐1, and miR‐92a‐1) (Tanzer & Stadler, 2004). All members of miR‐17‐92 cluster were overexpressed in colorectal cancer, pointing to a key role of miR‐17‐92 cluster in colorectal cancer carcinogenesis (Koga et al., 2010). It has been reported that the *MIR17HG *overexpression is associated with poor prognosis in colorectal cancer (Yu et al., 2012; Zhang, Li, Zhou, Xiao, & Zhou, 2014a; Zhou, Zhang, Liu, Xia, & Tian, 2013). Furthermore, functional studies have confirmed the pivotal role of members of the *MIR17HG* in the development, progression, and aggressiveness of colorectal cancer (Ma et al., 2016; Tsuchida et al., 2011; Zhang, Li, Zhou, et al., 2014a).

However, few association studies on polymorphisms of *MIR17HG* and colorectal cancer risk has been reported (Sun et al., 2017). To better understand the influence of *MIR17HG* polymorphisms on colorectal cancer susceptibility in the Chinese Han population. In this study, we recruited 514 patients with colorectal cancer and 510 healthy controls to investigate the association between polymorphisms (rs72640334, rs7336610, rs7318578, rs17735387, and rs1428) of *MIR17HG* and risk of colorectal cancer in the Chinese Han population.

## 2 MATERIALS AND METHODS

### 2.1 Study subjects

In this case–control study, we recruited of 514 colorectal cancer cases and 510 healthy control subjects from the Shaanxi Province Cancer Hospital. All cases were patients newly diagnosed with histologically confirmed colorectal cancer who were admitted to the hospital and without restrictions of age, sex, or disease stage. The patients who had received radiotherapy or chemotherapy were excluded in the study. The controls were randomly selected from the general health check‐up center at the same time period. The case and control subjects were unrelated ethnic Han Chinese and these subjects had no history of cancer. The characteristics of all subjects were taken from patients’ medical records by well‐trained interviewers.

### 2.2 Ethics statement

This study protocol was approved by the Ethics Committee of the Shaanxi Province Cancer Hospital and was conducted in accordance with the principles of the Declaration of Helsinki. All subjects provided written informed consent before the collection of blood samples.

### 2.3 DNA isolation

We used venipuncture into ethylene diamine tetraacetic acid‐coated blood vacutainer collection tubes to collect peripheral blood samples from each subject and then stored at −20°C for further DNA isolation. The GoldMag‐Mini Whole Blood Genomic DNA Purification Kit (GoldMag. Co. Ltd., Xi'an, China) was used to extract genomic DNA from blood samples following the manufacturer's instructions (Liu et al., 2017). The purity and concentration of the isolated DNA were analyzed using a NanoDrop 2000 spectrophotometer (Thermo Fisher Scientific, Waltham, MA) by absorbance measurements at 260 and 280 nm.

### 2.4 SNPs selection and genotyping

We selected the tagSNPs of *MIR17HG* with the minor allele frequency (MAF) greater than 0.05 in global population from the 1,000 Genome Projects. As a result, five tagSNPs (rs72640334, rs7336610, rs7318578, rs17735387, and rs1428) were selected using a pairwise Tagger method with *r* ^2^ > 0.8 to capture other SNPs. Primer sequences of amplification and extension for the polymorphisms of *MIR17HG* were designed using the Agena Bioscience Assay Design Suite V2.0 software (https://agenacx.com/online-tools/). Genotyping was performed with the Agena MassARRAY platform with iPLEX gold chemistry (Agena Bioscience, San Diego, CA) according to the standard protocol recommended by the manufacturer. Data management and analysis were performed using the Agena Bioscience TYPER software, version 4.0.

### 2.5 Statistical analysis

The differences between the cases and controls in demographic characteristics were evaluated by Student's *t *test (for age) and Pearson's *χ* ^2^ test (for gender). We used the chi‐square test to assess whether the genotype frequencies of SNPs among the control group was consistent with Hardy–Weinberg equilibrium (HWE). We compared the distributions of allele frequencies of SNPs between cases and controls using the *χ* ^2^ test. The association analyses were conducted under the codominant, dominant, recessive, and additive genetic models. Logistic regression analysis was carried out to calculate odds ratios (ORs) and its 95% confidence intervals (CIs) with the adjustment of gender and age (Dai et al., 2019). Pair‐wise linkage disequilibrium (LD) between the five SNPs was assessed using the Haploview software (version 4.2) (Barrett, Fry, Maller, & Daly, 2005). All two‐sided *p* values less than 0.05 were considered statistically significant. The statistical analyses were performed using the Statistical Package of the PLINK software (version 1.07) (Purcell et al., 2007) and Social Sciences (SPSS) software version 20.0 (SPSS Inc., Chicago, IL) and Microsoft Excel (Microsoft Corp., Redmond, WA).

## 3 RESULTS

### 3.1 Characteristics of study subjects

The demographic characteristics of participants are described in Table 1. Among the 1,024 participants, 514 were patients with colorectal cancer (228 females and 286 males) and 510 were healthy controls (224 females and 286 males). The mean age of the cases was 60.27 years old compared with 60.13 years old in controls, which revealed no statistically difference (*p* = 0.847). Furthermore, there was no significant difference in sex distribution *(p* = 0.839). Among the patients, the number of cases with colon cancer, rectal cancer, and other were 217 (42.2%), 244 (47.5%), and 53 (10.3%), respectively. The tumor stage for I–II, III–IV and missing were 146 (28.4%), 212 (41.2%), and 156 (30.4%), respectively.

**Table 1:** Basic characteristics of study objects

Characteristics		Case (%)	Control (%)	*p*
Number		514	510	
Age	Mean ± *SD* (years)	60.27 ± 11.81	60.13 ± 10.61	0.847
Gender	Female	228 (50.3)	224 (49.4)	0.839
	Male	286 (50.0)	286 (50.0)	
Tumor stage	I–II	146 (28.4)		
	III–V	212 (41.2)		
	Missing	156 (30.4)		
LN metastasis	No	188 (36.6)		
	Yes	170 (33.1)		
	Missing	156 (30.3)		
Tumor type	Colon cancer	217 (42.2)		
	Rectal cancer	244 (47.5)		
	Other	53 (10.3)		

Note

*p* values were calculated by Student's *t* test for age and *χ* ^2 ^test for gender.

*p* < 0.05 indicates statistical significance.

*SD*: standard deviation; LN: lymph node

### 3.2 Allele models analysis

Genotypes frequencies distributions of the five SNPs of *MIR17HG *among the healthy control were consistent with the HWE in this study, which indicated a representative distribution of the subjects in the regional population. The frequencies of rs7336610 minor allele T and rs1428 minor allele A among the patients with colorectal cancer were significantly different from those among the control subjects (*p* = 0.007; *p* = 0.008, respectively), as shown in Table 2. The results revealed that individuals carrying the allele T of rs7336610 and allele A of rs1428 were associated with significantly increased risk of colorectal cancer (OR = 1.27, 95% CI: 1.07–1.51; OR = 1.27, 95% CI: 1.06–1.51, respectively). However, no statistically significant association was found between the other four *MIR17HG* polymorphisms and colorectal cancer risk in the allele model.

**Table 2:** Association with between polymorphisms of *MIR17HG* and colorectal cancer risk

SNP‐ID	Chr	Position	Alleles A/B	MAF		OR (95% CI)	*p*
				Case	Control		
rs72640334	13	91352674	A/C	0.084	0.096	0.86 (0.64–1.17)	0.341
rs7336610	13	91352883	T/C	0.554	0.495	1.27 (1.07–1.51)	0.007
rs7318578	13	91353215	C/A	0.263	0.297	0.85 (0.70–1.03)	0.089
rs17735387	13	91353800	A/G	0.164	0.188	0.85 (0.68–1.07)	0.157
rs1428	13	91354516	A/C	0.555	0.496	1.27 (1.06–1.51)	0.008

Note

SNP: Single nucleotide polymorphism; Chr: chromosome; A: Minor alleles; B: Major alleles; MAF: Minor allele frequency; OR: Odds ratio; 95% CI: 95% Confidence interval

*p* values were calculated from *χ* ^2^ test ( two sided).

*p < *0.05 was considered statistically significant.

### 3.3 Genetic models analysis

Then, we further assessed the association between the five SNPs of *MIR17HG *and colorectal cancer risk under the four genetic models (codominant, dominant, recessive, and additive) by logistic regression analysis adjusting for gender and age (Table 3). Compared to the wild homozygous genotype CC of rs7336610, individuals carrying rs7336610 TT genotype were associated with a significantly increased risk of colorectal cancer before and after adjusting for gender and age (OR = 1.57, 95% CI: 1.12–2.20, *p* = 0.009; Table 3). The SNP rs7336610 was also found to be associated with an increased risk of colorectal cancer in the dominant model (TT + TC vs. CC: OR = 1.44, 95% CI: 1.08–1.92, *p* = 0.013) and the additive model (OR = 1.25, 95% CI: 1.06–1.48, *p* = 0.010) before and after adjusting for gender and age. However, we found rs7318578 was associated a decreased risk of colorectal cancer in the dominant model before adjusting for gender and age (CC + CA vs. AA: OR = 0.78, 95% CI: 0.61–1.00, *p* = 0.049).

**Table 3:** Genetics model analysis of association between *MIR17HG* polymorphisms and colorectal cancer risk

SNP‐ID	Model	Genotype	Case	Control	OR (95% CI)	*p*	Adjusted OR (95% CI)	Adjusted *p*
rs72640334	Codominant	CC	426	416	1.00		1.00	
		CA	75	90	0.81 (0.58–1.14)	0.228	0.82 (0.58–1.14)	0.234
		AA	5	4	1.22 (0.33–4.58)	0.768	1.23 (0.33–4.60)	0.763
	Dominant	CC	426	416	1.00		1.00	
		AA + CA	80	94	0.83 (0.60–1.15)	0.268	0.83 (0.60–1.16)	0.275
	Recessive	CC + CA	501	506	1.00		1.00	
		AA	5	4	1.26 (0.34–4.73)	0.729	1.27 (0.34–4.75)	0.725
	Additive	–	–	–	0.86 (0.64–1.17)	0.344	0.87 (0.64–1.17)	0.352
rs7336610	Codominant	CC	109	142	1.00		1.00	
		TC	240	230	1.36 (1.00–1.85)	0.051	1.36 (1.00–1.85)	0.051
		TT	165	137	1.57 (1.12–2.20)	0.009	1.57 (1.12–2.20)	0.009
	Dominant	CC	109	142	1.00		1.00	
		TT + TC	405	367	1.44 (1.08–1.92)	0.013	1.44 (1.08–1.92)	0.013
	Recessive	CC + TC	349	372	1.00		1.00	
		TT	165	137	1.28 (0.98–1.68)	0.069	1.28 (0.98–1.68)	0.070
	Additive	–	–	–	1.25 (1.06–1.48)	0.010	1.25 (1.06–1.48)	0.010
rs7318578	Codominant	AA	277	245	1.00		1.00	
		CA	199	227	0.78 (0.60–1.00)	0.052	0.78 (0.60–100)	0.054
		CC	35	38	0.81 (0.50–1.33)	0.413	0.82 (0.50–1.33)	0.417
	Dominant	AA	277	245	1.00		1.00	
		CC + CA	234	265	0.78 (0.61–1.00)	0.049	0.78 (0.61–1.00)	0.051
	Recessive	AA + CA	476	472	1.00		1.00	
		CC	35	38	0.91 (0.57–1.47)	0.709	0.91 (0.57–1.47)	0.713
	Additive	–	–	–	0.84 (0.69–1.02)	0.083	0.84 (0.69–1.03)	0.087
rs17735387	Codominant	GG	361	335	1.00		1.00	
		GA	137	158	0.80 (0.61–1.06)	0.119	0.80 (0.61–1.06)	0.116
		AA	16	17	0.87 (0.43–1.76)	0.704	0.87 (0.43–1.75)	0.695
	Dominant	GG	361	335	1.00		1.00	
		AA + GA	153	175	0.81 (0.62–1.06)	0.119	0.81 (0.62–1.05)	0.116
	Recessive	GG + GA	498	493	1.00		1.00	
		AA	16	17	0.93 (0.47–1.87)	0.842	0.93 (0.46–1.87)	0.837
	Additive	–	–	–	0.85 (0.68–1.07)	0.159	0.85 (0.67–1.07)	0.155
rs1428	Codominant	CC	109	142	1.00		1.00	
		CA	239	228	1.37 (1.00–1.86)	0.048	1.37 (1.00–1.86)	0.048
		AA	165	138	1.56 (1.11–2.18)	0.010	1.56 (1.11–2.19)	0.010
	Dominant	CC	109	142	1.00		1.00	
		AA + CA	404	366	1.44 (1.08–1.92)	0.013	1.44 (1.08–1.92)	0.013
	Recessive	CC + CA	348	370	1.00		1.00	
		AA	165	138	1.27 (0.97–1.66)	0.081	1.27 (0.97–1.67)	0.081
	Additive	–	–	–	1.24 (1.05–1.47)	0.011	1.24 (1.05–1.47)	0.011

Note

SNP: Single nucleotide polymorphism; OR: Odds ratio; 95% CI: 95% Confidence interval

*p* < 0.05 was considered statistically significant.

In addition, the genotypes CA and AA of rs1428 were significantly associated with increased risk of colorectal cancer, compared to the CC genotype before and after adjusting for gender and age (CA vs. CC: OR = 1.37, 95% CI: 1.00–1.86, *p* = 0.048; AA vs. CC: OR = 1.56, 95% CI: 1.11–2.19, *p* = 0.010). In both the dominant and additive models, there were significant association between rs1428 and the risk of colorectal cancer before and after adjusting for gender and age (dominant AA + CA vs. CC: OR = 1.44, 95% CI: 1.08–1.92, *p* = 0.013; additive: OR = 1.24, 95% CI: 1.05–1.47, *p* = 0.011). However, no significant association between the other SNPs of *MIR17HG *(rs72640334 and rs17735387) and colorectal cancer risk under the four genetic models.

### 3.4 Tumor type stratification analysis

Statistical analysis based on stratification of tumor type revealed that the SNP rs7336610 was remarkably increased risk of rectal cancer after adjusting for gender and age (allele T vs. C: OR = 1.32, 95% CI: 1.06–1.64, *p* = 0.013; TC vs. CC: OR = 1.56, 95% CI: 1.05–2.33, *p* = 0.028; TT vs. CC: OR = 1.72, 95% CI: 1.12–2.66, *p* = 0.014; dominant TT + TC vs. CC: OR = 1.62, 95% CI: 1.12–2.36, *p* = 0.011; additive: OR = 1.30, 95% CI: 1.05–1.60, *p* = 0.017) (Table 4). The SNP rs1428 was also found to be associated with a higher risk of rectal cancer after adjusting for gender and age (allele A vs. C: OR = 1.31, 95% CI: 1.06–1.63, *p* = 0.014; CA vs. CC: OR = 1.57, 95% CI: 1.05–2.33, *p* = 0.027; dominant AA + CA vs. CC: OR = 1.62, 95% CI: 1.12–2.36, *p* = 0.011; additive: OR = 1.29, 95% CI: 1.05–1.60, *p* = 0.018). The SNP rs7318578 had a significantly lower risk of rectal cancer (CA vs. AA: OR = 0.71, 95% CI: 0.51–0.98, *p* = 0.038; dominant CC + CA vs. AA: OR = 0.73, 95% CI: 0.54–1.00, *p* = 0.047). However, no association was found between the SNPs of *MIR17HG* and colon cancer risk.

**Table 4:** Tumor type stratification analysis of association between *MIR17HG* polymorphisms and colorectal cancer risk

SNP–ID	Model	Genotype	Rectal cancer			Colon cancer				
			Case	Control	OR (95% CI)	*p*	Case	Control	OR (95% CI)	*p*
rs7336610	Allele	C	213	514	1.00		199	514	1.00	
		T	275	504	1.32 (1.06–1.64)	0.013	235	504	1.20 (0.96–1.51)	0.106
	Codominant	CC	47	142	1.00		50	142	1.00	
		TC	119	230	1.56 (1.05–2.33)	0.028	99	230	1.23 (0.82–1.84)	0.310
		TT	78	137	1.72 (1.12–2.66)	0.014	68	137	1.42 (0.92–2.19)	0.119
	Dominant	CC	47	142	1.00		50	142	1.00	
		TT + TC	197	367	1.62 (1.12–2.36)	0.011	167	367	1.30 (0.90–1.89)	0.168
	Recessive	CC + TC	166	372	1.00		149	372	1.00	
		TT	78	137	1.28 (0.91–1.78)	0.152	68	137	1.24 (0.87–1.75)	0.229
	Additive	–	–	–	1.30 (1.05–1.60)	0.017	–	–	1.19 (0.96–1.47)	0.120
rs7318578	Allele	A	359	717	1.00		311	717	1.00	
		C	125	303	0.82 (0.65–1.05)	0.119	121	303	0.92 (0.72–1.18)	0.516
	Codominant	AA	135	245	1.00		111	245	1.00	
		CA	89	227	0.71 (0.51–0.98)	0.038	89	227	0.88 (0.63–1.22)	0.434
		CC	18	38	0.86 (0.47–1.56)	0.618	16	38	0.93 (0.50–1.75)	0.828
	Dominant	AA	135	245	1.00		111	245	1.00	
		CC + CA	107	283	0.73 (0.54–1.00)	0.047	105	265	0.88 (0.64–1.22)	0.449
	Recessive	AA + CA	224		1.00		200	472	1.00	
		CC	18	38	1.00 (0.56–1.79)	0.994	16	38	0.99 (0.54–1.82)	0.979
	Additive	–	–	–	0.82 (0.64–1.05)	0.113	–	–	0.92 (0.71–1.19)	0.538
rs1428	Allele	C	212	512	1.00		199	512	1.00	
		A	274	504	1.31 (1.06–1.63)	0.014	235	504	1.20 (0.96–1.50)	0.113
	Codominant	CC	47	142	1.00		50	142	1.00	
		CA	118	228	1.57 (1.05–2.33)	0.027	99	228	1.24 (0.83–1.86)	0.288
		AA	78	138	1.71 (1.11–2.64)	0.015	68	138	1.41 (0.91–2.17)	0.125
	Dominant	CC	47	142	1.00		50	142	1.00	
		AA + CA	196	366	1.62 (1.12–2.36)	0.011	167	366	1.30 (0.90–1.89)	0.162
	Recessive	CC + CA	165	370	1.00		149	370	1.00	
		AA	78	138	1.27 (0.91–1.77)	0.161	68	138	1.22 (0.86–1.73)	0.256
	Additive	–	–	–	1.29 (1.05–1.60)	0.018	–	–	1.18 (0.95–1.47)	0.127

Note

SNP: Single nucleotide polymorphism; OR: Odds ratio; 95% CI: 95% Confidence interval

OR and 95% CI were calculated using logistic regression adjusted with age and gender.

*p* < 0.05 was considered statistically significant.

### 3.5 Gender stratification analysis

Gender stratification analysis found rs7336610 (allele T vs. C: OR = 1.33, 95% CI: 1.06–1.68, *p* = 0.015; dominant TT + TC vs. CC: OR = 1.48, 95% CI: 1.02–2.15, *p* = 0.039; additive: OR = 1.30, 95% CI: 1.04–1.63, *p* = 0.021) and rs1428 (allele A vs. C: OR = 1.33, 95% CI: 1.06–1.68, *p* = 0.015; AA vs. CC: OR = 1.70, 95% CI: 1.08–2.65, *p* = 0.021; dominant AA + CA vs. CC: OR = 1.50, 95% CI: 1.03–2.17, *p* = 0.034; additive: OR = 1.30, 95% CI: 1.04–1.63, *p* = 0.021) were significantly associated with increased risk of colorectal cancer after adjusting for age in males (Table 5). Moreover, rs7318578 (CA vs. AA: OR = 0.66, 95% CI: 0.47–0.94, *p* = 0.020; dominant CC + CA vs. AA: OR = 0.70, 95% CI: 0.51–0.98, *p* = 0.036) and rs17735387 (GA vs. GG: OR = 0.67, 95% CI: 0.46–0.96, *p* = 0.031) were found to be associated with reduced risk of colorectal cancer after adjusting for age in males. However, no association was found between the SNPs of *MIR17HG* and colorectal cancer risk among females.

**Table 5:** Gender stratification analysis of association between *MIR17HG* polymorphisms and colorectal cancer risk

SNP‐ID	Model	Genotype	Male				Female			
			Case	Control	OR (95% CI)	*p*	Case	Control	OR (95% CI)	*p*
rs7336610	Allele	C	263	304	1.00		261	236	1.00	
		T	309	268	1.33 (1.06–1.68)	0.015	195	210	0.84 (0.65–1.09)	0.192
	Codominant	CC	66	88	1.00		76	67	1.00	
		TC	131	128	1.36 (0.91–2.04)	0.130	109	102	0.94 (0.61–1.44)	0.770
		TT	89	70	1.70 (1.08–2.65)	0.021	43	54	0.70 (0.42–1.18)	0.177
	Dominant	CC	66	88	1.00		76	67	1.00	
		TT + TC	220	198	1.48 (1.02–2.15)	0.039	152	156	0.86 (0.58–1.27)	0.443
	Recessive	CC + TC	197	216	1.00		185	169	1.00	
		TT	89	70	1.39 (0.97–2.01)	0.077	43	54	0.73 (0.46–1.14)	0.166
	Additive	–	–	–	1.30 (1.04–1.63)	0.021	–	–	0.85 (0.65–1.09)	0.199
rs7318578	Allele	A	413	394	1.00		340	323	1.00	
		C	155	178	0.83 (0.64–1.07)	0.155	114	125	0.87 (0.64–1.17)	0.342
	Codominant	AA	152	128	1.00		125	117	1.00	
		CA	109	138	0.66 (0.47–0.94)	0.020	90	89	0.95 (0.65–1.40)	0.803
		CC	23	20	0.97 (0.51–1.84)	0.920	12	18	0.62 (0.29–1.35)	0.231
	Dominant	AA	152	128	1.00		125	117	1.00	
		CC + CA	132	158	0.70 (0.51–0.98)	0.036	102	107	0.90 (0.62–1.30)	0.563
	Recessive	AA + CA	261	266	1.00		215	206	1.00	
		CC	23	20	1.17 (0.63–2.19)	0.618	12	18	0.64 (0.30–1.36)	0.242
	Additive	–	–	–	0.82 (0.63–1.07)	0.145	–	–	0.87 (0.64–1.17)	0.346
rs17735387	Allele	G	477	457	1.00		382	371	1.00	
		A	95	115	0.79 (0.59–1.07)	0.127	74	77	0.93 (0.66–1.32)	0.699
	Codominant	GG	203	181	1.00		158	154	1.00	
		GA	71	95	0.67 (0.46–0.96)	0.031	66	63	1.02 (0.67–1.53)	0.944
		AA	12	10	1.07 (0.45–2.54)	0.876	4	7	0.54 (0.15–1.88)	0.331
	Dominant	GG	203	181	1.00		158	154	1.00	
		AA + GA	83	105	0.70 (0.50–1.00)	0.051	70	70	0.97 (0.65–1.44)	0.872
	Recessive	GG + GA	274	276	1.00		224	217	1.00	
		AA	12	10	1.21 (0.51–2.86)	0.660	4	7	0.53 (0.15–1.86)	0.325
	Additive	–	–	–	0.80 (0.59–1.07)	0.135	–	–	0.92 (0.65–1.32)	0.661
rs1428	Allele	C	263	302	1.00		260	238	1.00	
		A	309	266	1.33 (1.06–1.68)	0.015	194	210	0.85 (0.65–1.10)	0.211
	Codominant	CC	66	88	1.00		76	68	1.00	
		CA	131	126	1.39 (0.93–2.07)	0.112	108	102	0.94 (0.62–1.44)	0.788
		AA	89	70	1.70 (1.08–2.65)	0.021	43	54	0.71 (0.42–1.19)	0.194
	Dominant	CC	66	88	1.00		76	68	1.00	
		AA + CA	220	196	1.50 (1.03–2.17)	0.034	151	156	0.86 (0.58–1.28)	0.464
	Recessive	CC + CA	197	214	1.00		184	170	1.00	
		AA	89	70	1.38 (0.96–2.00)	0.086	43	54	0.73 (0.47–1.15)	0.181
	Additive	–	–	–	1.30 (1.04–1.63)	0.021	–	–	0.85 (0.66–1.10)	0.218

Note

SNP: Single nucleotide polymorphism; OR: Odds ratio; 95% CI: 95% Confidence interval

OR and 95% CI were calculated using logistic regression adjusted with age and gender.

*p* < 0.05 was considered statistically significant.

### 3.6 LD and haplotype analysis

**Figure 1:**
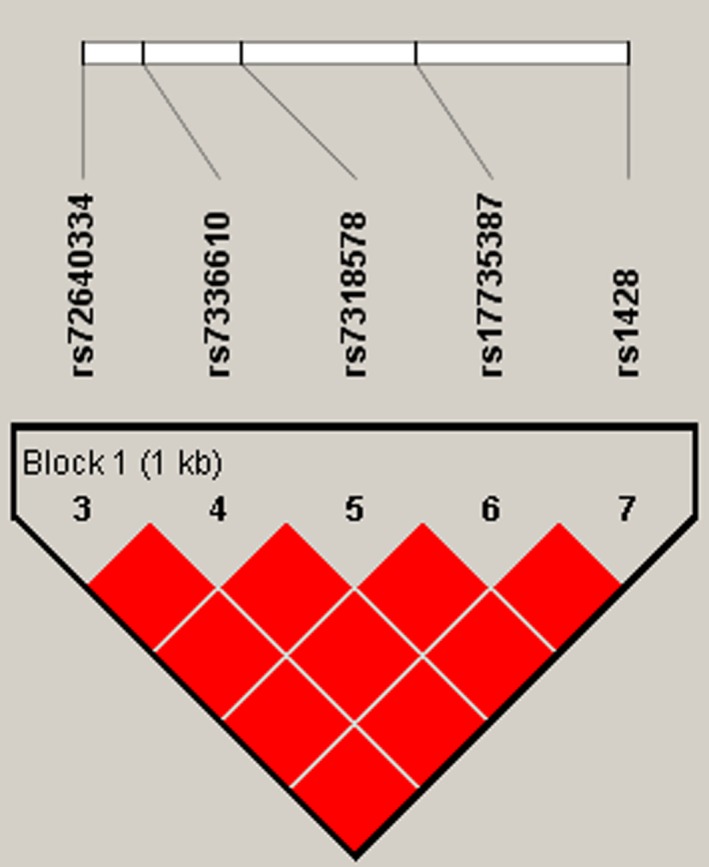
Haplotype block map for the five SNPs in the *MIR17HG* gene. Standard color frame is used to show LD pattern. One block in the figure showed higher LD. Bright red represents very strong LD

The results of pair‐wise LD analysis with these five SNPs are shown in Figure 1. We observed one haplotype block composed of rs72640334, rs7336610, rs7318578, rs17735387, and rs1428. Overall analysis found that the distributions of the frequency of the haplotype CTAGA were significantly different between colorectal cancer and control groups (*p* = 0.007); and the haplotype CTAGA was significantly associated with an increased colorectal cancer risk after adjusting for gender and age (OR = 1.26, 95% CI: 1.06–1.49) (Table 6). Statistical analysis found that the haplotype CTAGA was also associated with high risk of rectal cancer after adjusting for gender and age (OR = 1.29, 95% CI: 1.04–1.59, *p* = 0.018). Furthermore, in logistic regression analysis adjusted for age and gender, the haplotype CTAGA in *MIR17HG* was associated with high risk of colorectal cancer among males (OR = 1.32, 95% CI: 1.06–1.65, *p* = 0.015).

**Table 6:** Haplotype analysis of association between *MIR17HG *polymorphisms and colorectal cancer risk

SNP‐ID	Haplotype	Overall				Rectal cancer				Male			
		Case	Control	OR (95% CI)	*p*	Case	Control	OR (95% CI)	*p*	Case	Control	OR (95% CI)	*p*
rs72640334|	CTAGA	0.558	0.496	1.26 (1.06–1.49)	0.007	0.564	0.496	1.29 (1.04–1.59)	0.018	0.544	0.469	1.32 (1.06–1.65)	0.015
rs7336610|	CCAAC	0.834	0.812	1.17 (0.93–1.47)	0.183	0.846	0.812	1.27 (0.95–1.70)	0.113	0.832	0.799	1.24 (0.92–1.67)	0.160
rs7318578|	ACCGC	0.917	0.904	1.17 (0.86–1.58)	0.323	0.918	0.904	1.18 (0.81–1.73)	0.394	0.905	0.897	1.09 (0.74–1.61)	0.657
rs17735387|	CCCGC	0.824	0.799	1.18 (0.94–1.49)	0.148	0.823	0.799	1.19 (0.89–1.58)	0.250	0.827	0.792	1.28 (0.94–1.75)	0.118
rs1428	CCAGC	0.983	0.981	1.12 (0.58–2.18)	0.739	0.977	0.981	0.82 (0.38–1.74)	0.598	0.981	0.981	0.99 (0.42–2.32)	0.980

Note

SNP: Single nucleotide polymorphism; OR: Odds ratio; 95% CI: 95% Confidence interval

OR and 95% CI were calculated using logistic regression adjusted with age and gender.

*p* < 0.05 was considered statistically significant.

## 4 DISCUSSION

In this case–control study, we investigated the association between the polymorphisms of *MIR17HG* and colorectal cancer risk in the Chinese Han population. Overall analysis indicated that rs7336610 and rs1428 were associated with increased risk of colorectal cancer; but rs7318578 was associated with a decreased risk of colorectal cancer under the dominant model. Stratification analysis showed that rs7336610, rs7318578, rs17735387, and rs1428 were associated with colorectal cancer risk. Moreover, haplotype analysis confirmed that the haplotype CTAGA was significantly associated with an increased risk of colorectal cancer.

**Figure 2:**
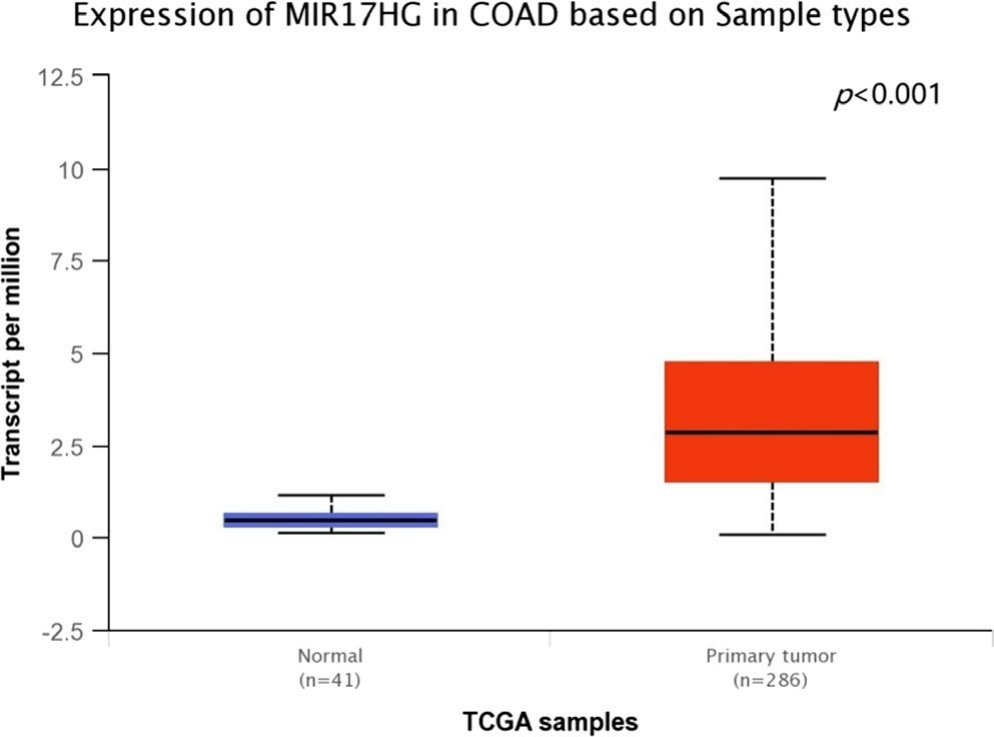
The expression of *MIR17HG* between normal and colon adenocarcinoma tissues from the UALCAN database. COAD: colon adenocarcinoma

**Figure 3:**
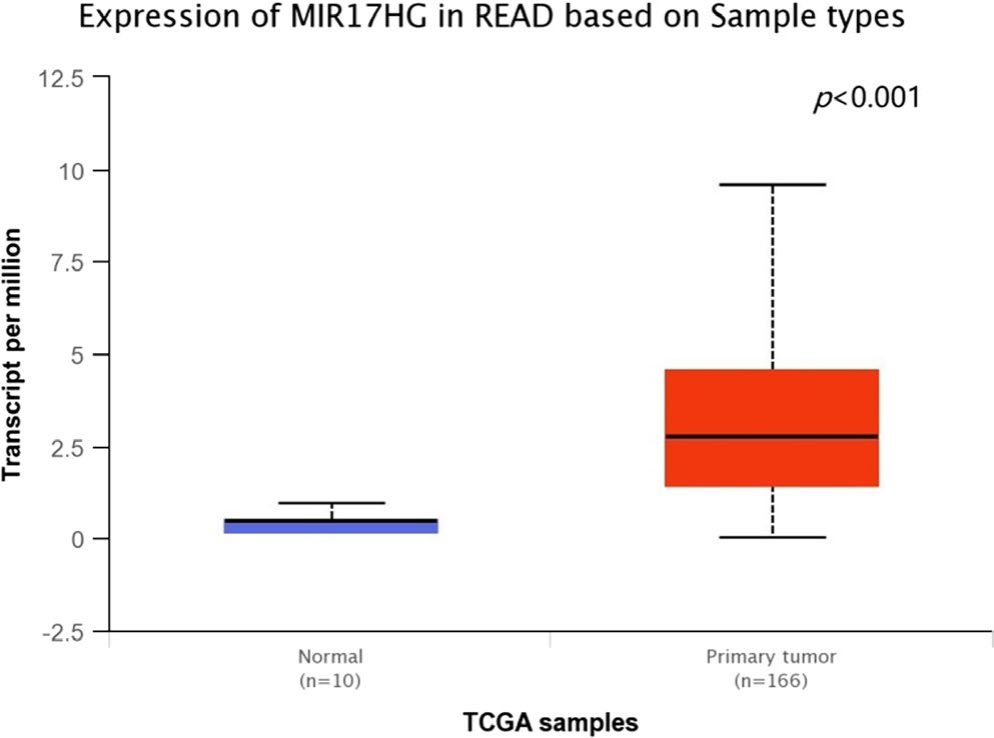
The expression of *MIR17HG* between normal and rectum adenocarcinoma tissues from the UALCAN database. READ: Rectum adenocarcinoma

*MIR17HG* is located at humans chromosome 13q31, a genomic region frequently amplified in a large spectrum of human cancers including colorectal cancer. According to UALCAN database (http://ualcan.path.uab.edu/cgi-bin/ualcan-res.pl), we found that the expression of *MIR17HG *is significantly different between normal and colon and rectal cancer tissues (Figures 2 and 3; Chandrashekar et al., 2017). The overexpression of miR‐17‐92 cluster is not only involved in the progression of colorectal adenoma to adenocarcinoma but also related to poor survival of colorectal cancer (Diosdado et al., 2009; Yu et al., 2012). Previous study demonstrated that miR‐17‐92 suppressed colorectal cancer progression by inhibiting tumor angiogenesis in a genetically engineered mouse model, indicating the presence of cellular context‐dependent pro‐ and anti‐cancer effects of miR‐17‐92 (Ma et al., 2016). It also found that higher levels of miR‐17‐92 contribute to inhibition of tumor growth and metastasis in a mouse tumor model (Jiang et al., 2014). Recent research identified that the miR‐17‐92 cluster was a crucial player in the development of the immune system (Kuo, Wu, & Yang, 2018). Previous study indicated that *MIR17HG* copy numbers would seem to be related to response to neoadjuvant chemoradiotherapy in locally advanced rectal cancer (Molinari et al., 2016). Moreover, it has been reported that the miR‐92 upregulation in plasma may be used as a noninvasive molecular marker for colorectal cancer screening, with a sensitivity of 89% and a specificity of 70% (Ng et al., 2009). These findings suggest that miR‐17‐92 cluster play a pivotal role in the of development colorectal cancer.

Previous study reported that two functional polymorphisms (rs9588884 and rs982873) in the promoter region of miR‐17‐92 cluster are associated with a decreased risk of colorectal cancer (Sun et al., 2017). It has been reported that the SNP rs9515692 in the promoter region of miR‐17‐92 was a protective factor for the susceptibility of systemic lupus erythematosus (Wang et al., 2018). Statistical analysis of allele frequencies in cases and controls in the Genomics Research Centre Breast Cancer population for rs7336610 showed significance; and haplotypic analysis of results showed that the AC haplotype of rs4824505/rs7336610 are associated with risk of breast cancer development (Chacon‐Cortes, Smith, Lea, Youl, & Griffiths, 2015). In this study, we investigated the association between the polymorphisms of *MIR17HG* and colorectal cancer risk in the Chinese Han population. The results indicated that the two SNPs (rs7336610 and rs1428) of *MIR17HG *were associated with increased colorectal cancer risk, but the two SNPs (rs7318578, rs17735387) of *MIR17HG* were associated with decreased colorectal cancer risk in the Chinese Han population. To date, no association study was carried out to investigate the association of SNPs (rs72640334, rs7336610, rs7318578, rs17735387, and rs1428) of *MIR17HG* and colorectal cancer risk. Therefore, further association study with a large sample is needed to confirm these results.

There are some potential limitations in this study must be considered. First, only subjects of Chinese Han descent were included in this study, additional studies included different ethnic populations should be conducted to confirm these results. Second, data were not available for some risk factors (e.g., cigarette smoking, alcohol consumption), which prevented our further gene‐environment interaction analysis. Third, functional studies were not performed in this study. More detailed data are required to create a comprehensive understanding of the *MIR17HG* in colorectal cancer tumorigenesis.

## 5 CONCLUSIONS

In conclusion, this study provides the first evidence that the polymorphisms (rs7336610, rs7318578, rs17735387, and rs1428) of *MIR17HG* were associated with colorectal cancer risk. Therefore, our findings may provide new insights into the development of colorectal cancer. Further association and functional studies are of great importance to confirm these results and help us to define the potential biological mechanism of colorectal cancer.

## CONFLICT OF INTEREST

The authors declare that there are no conflicts of interest.
